# Cardiovascular magnetic resonance myocardial feature tracking detects quantitative wall motion during dobutamine stress

**DOI:** 10.1186/1532-429X-13-58

**Published:** 2011-10-12

**Authors:** Andreas Schuster, Shelby Kutty, Asif Padiyath, Victoria Parish, Paul Gribben, David A Danford, Marcus R Makowski, Boris Bigalke, Philipp Beerbaum, Eike Nagel

**Affiliations:** 1 King's College London British Heart Foundation (BHF) Centre of Excellence; National Institute of Health Research (NIHR) Biomedical Research Centre at Guy's and St. Thomas' NHS Foundation Trust; Wellcome Trust and Engineering and Physical Sciences Research Council (EPSRC) Medical Engineering Centre; Division of Imaging Sciences and Biomedical Engineering; The Rayne Institute, St. Thomas' Hospital, London, UK; 2 Joint Division of Pediatric Cardiology, University of Nebraska/Creighton University, Children's Hospital and Medical Center, Omaha, USA; 3 Evelina Children's Hospital, Department of Paediatric Cardiology, Guy's and St. Thomas' NHS Foundation Trust, London, UK; 4 Department of Radiology, Charite, Universitätsmedizin, Berlin, Germany; 5 Medizinische Klinik III, Kardiologie und Kreislauferkrankungen, Eberhard-Karls-Universitt Tbingen, Tbingen, Germany

## Abstract

**Background:**

Dobutamine stress cardiovascular magnetic resonance (DS-CMR) is an established tool to assess hibernating myocardium and ischemia. Analysis is typically based on visual assessment with considerable operator dependency. CMR myocardial feature tracking (CMR-FT) is a recently introduced technique for tissue voxel motion tracking on standard steady-state free precession (SSFP) images to derive circumferential and radial myocardial mechanics.

We sought to determine the feasibility and reproducibility of CMR-FT for quantitative wall motion assessment during intermediate dose DS-CMR.

**Methods:**

10 healthy subjects were studied at 1.5 Tesla. Myocardial strain parameters were derived from SSFP cine images using dedicated CMR-FT software (Diogenes MRI prototype; Tomtec; Germany). Right ventricular (RV) and left ventricular (LV) longitudinal strain (Ell_RV _and Ell_LV_) and LV long-axis radial strain (Err_LAX_) were derived from a 4-chamber view at rest. LV short-axis circumferential strain (Ecc_SAX_) and Err_SAX_; LV ejection fraction (EF) and volumes were analyzed at rest and during dobutamine stress (10 and 20 μg · kg^-1^· min^-1^).

**Results:**

In all volunteers strain parameters could be derived from the SSFP images at rest and stress. Ecc_SAX _values showed significantly increased contraction with DSMR (rest: -24.1 ± 6.7; 10 μg: -32.7 ± 11.4; 20 μg: -39.2 ± 15.2; p < 0.05). Err_SAX _increased significantly with dobutamine (rest: 19.6 ± 14.6; 10 μg: 31.8 ± 20.9; 20 μg: 42.4 ± 25.5; p < 0.05). In parallel with these changes; EF increased significantly with dobutamine (rest: 56.9 ± 4.4%; 10 μg: 70.7 ± 8.1; 20 μg: 76.8 ± 4.6; p < 0.05). Observer variability was best for LV circumferential strain (Ecc_SAX _) and worst for RV longitudinal strain (Ell_RV_) as determined by 95% confidence intervals of the difference.

**Conclusions:**

CMR-FT reliably detects quantitative wall motion and strain derived from SSFP cine imaging that corresponds to inotropic stimulation. The current implementation may need improvement to reduce observer-induced variance. Within a given CMR lab; this novel technique holds promise of easy and fast quantification of wall mechanics and strain.

## Background

Cardiovascular magnetic resonance (CMR) plays an increasingly important role in the diagnosis and assessment of coronary artery disease (CAD). It has evolved into a comprehensive clinical tool with the unique capability of assessing myocardial function; viability and perfusion in a single examination [1].

Wall motion analysis with CMR has a pivotal role in clinical practice. It is considered the gold standard for visualizing left ventricular (LV) endocardial wall motion at rest; as well as during low and high dose dobutamine stress to assess myocardial hibernation and ischemia. At the present time; image analysis is most commonly performed qualitatively. However diagnostic accuracy of qualitative assessment has been shown to be considerably operator dependant [2].

Deformation assessment of tagged lines within the myocardium may overcome these limitations however requires acquisition of additional tagging sequences and post processing [3]. Recently CMR myocardial feature tracking (FT); a technique analogous to echocardiographic speckle tracking; has been introduced [4]. CMR-FT allows tracking of tissue voxel motion of cine-CMR images with a potential to assess longitudinal; circumferential and radial myocardial strain as well as velocity; displacement and torsion independent of additional sequences. A good agreement of CMR-FT versus myocardial tagging with harmonic phase imaging (HARP) as a reference standard has been demonstrated [5]. However it is unclear; whether this approach would allow the response to dobutamine stress to be quantified [6]. The aim of the current study was to determine the ability of CMR-FT for quantitative wall motion assessment at rest and during intermediate dose dobutamine stress in healthy volunteers.

## Methods

Ten healthy volunteers underwent CMR on a 1.5 Tesla scanner (Intera R 12.6.1.3; Philips Medical Systems; Best; The Netherlands). The study protocol was approved by the Institutional Review Board at the University of Nebraska Medical Center. All participants gave written informed consent.

### Cardiovascular magnetic resonance

All CMR measurements were performed in the supine position using a 5-channel cardiac surface coil. LV dimensions and function were assessed with an ECG-gated steady state free-precession cine sequence during brief periods of breath-holding in the following planes: ventricular 2-chamber; 4-chamber; and 12 to 14 equidistant short-axis planes (slice thickness 6-8 mm; gap 0-2 mm) completely covering both ventricles. The field of view was 360 × 480 mm and matrix size 196 × 172. Dobutamine stress imaging was performed as previously described [7]. Repeat short-axis stacks were acquired with 10 and 20 μg · kg^-1^· min^-1 ^of dobutamine; respectively.

### Ventricular volumes and function

End-diastolic (EDV) and end-systolic volumes (ESV); stroke volume (SV); and ejection fraction (EF) were measured as previously described using commercially available software packages (View Forum; Philips) [8]. Ventricular volumes were adjusted to body surface area. All parameters were analysed at rest; 10 and 20 μg · kg^-1^· min^-1 ^of dobutamine stress.

### Feature tracking

**Figure 1:**
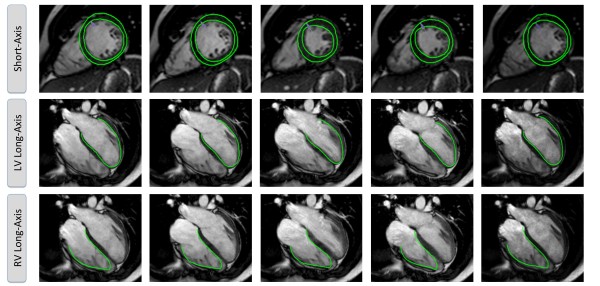
**Tracking in Short-Axis and Long-Axis Orientation**. The figure shows a representative example of the tracking in Short-Axis and Long-Axis Orientation of the left ventricle (LV) and the right ventricle (RV).

CMR-FT analysis of strain was performed using a dedicated software prototype (Diogenes MRI; Tomtec; Germany). The 4-chamber view was used to calculate right ventricular (RV) and LV longitudinal strain and LV radial strain (Ell_RV _and Ell_LV _and Err_LAX_) at rest. LV short axis circumferential (Ecc_SAX_) and radial strains (Err_SAX_) were derived from a mid-ventricular short-axis view containing both papillary muscles. The RV upper septal insertion point of the LV was manually detected to allow accurate segmentation according to a recognized standard model [9]. Endocardial contours were manually drawn in all analyzed slices by one skilled observer (AS; 7 years of experience). Ecc_SAX _and Err_SAX _were analysed at rest; 10 and 20 μg · kg^-1^· min^-1 ^of dobutamine stress. A second observer (SK; 4 years of experience) re-analysed the images to assess inter-observer variability. The mid-ventricular short axis images analyzed by the second observer were at exactly the same slice position as for the first observer. The first observer repeated the measurements after a period of 4 weeks to assess intra-observer variability. Figure 1 shows a representative example of the tracking of LV and RV in the respective views.

### Comparison with natural radial strain

Natural radial strain values were obtained as an external reference standard and compared to the respective CMR-FT Err_SAX _values [10]. In brief end-diastolic and end-systolic wall-thickness (EDWT and ESWT) were quantified in identical segments as analysed for Err_SAX _using commercially available software (Philips View Forum; The Netherlands) [11]. Natural radial strain values were calculated according to the following equation: *log_e _(ESWT/EDWT) *as previously validated [10]. 95% confidence intervalls of the difference and p-values were calculated to compare the 2 techniques [11].

### Statistics

We have applied a paired t-test followed by a Bonferroni-Holm correction as a multiple test procedure to compare measurements at rest and with dobutamine stress after proving a normal distribution of the sample. Intra- and inter-observer variability analysis were performed using the method proposed by Bland and Altman [12]. A p-value of <0.05 was considered statistically significant. All data analysis was performed with PASW statistics for Mac 18.0.0 (SPSS Inc.; Chicago; Illinois; USA).

## Results

The image quality was sufficient to perform strain analysis in all segments for all subjects. Gender was equally distributed and LV and RV volumes were within normal limits [13]. Participant demographics are shown in table 1. There were no side effects to dobutamine exposure. There was significant (p < 0.05) increase of heart rate; mean blood pressure and cardiac output between rest and both levels of dobutamine as well as between 10 and 20 μg · kg^-1^· min^-1 ^of dobutamine

**Table 1:** Subject Characteristics

Demographics	"Normal" Healthy Volunteers
Study population	N = 10
Gender	Male 50%, Female 50%
Age (y)	40.6 (23.9-51.8)
RV-EDV (ml/m^2^)	76.6 ± 14.3
RV-ESV(ml/m^2^)	32.1 ± 8.6
RV-CI (l/min/m^2^)	3.0 ± 0.6
RV-EF (%)	58.5 ± 4.1
Ell_RV_	-19.7 ± 14.1
LV-EDV (ml/m^2^)	76.9 ± 12.5
LV-ESV (ml/m^2^)	33.4 ± 7.5
LV-CI (l/min/m^2^)	3.0 ± 0.6
LV-EF (%)	56.9 ± 4.4
Ell_LV_	-15.9 ± 10.5
Err_LAX_	15.3 ± 10.1
Err_SAX_	19.6 ± 14.6
Ecc_SAX_	-24.1 ± 6.7

Continuous variable are expressed as mean ± standard deviation, age is expressed as median with range. *RV: right ventricle, LV left ventricle, EDV: enddiastolic volume, ESV: endsystolic volume, Ell_RV _= right ventricular longitudinal strain, Ell_LV _= left ventricular longitudinal strain, Err_LAX _= left ventricular long-axis radial strain, Err_SAX _= left ventricular short-axis radial strain, Ecc_SAX _= left ventricular short-axis circumferential strain, EF = ejection fraction*

### Strain parameters at rest

Results at rest are displayed in table 1.

### Dobutamine stress cardiovascular magnetic resonance

**Figure 2:**
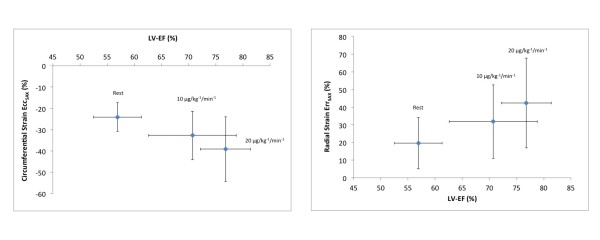
**Circumferential and radial strain in respect to changes of left ventricular ejection fraction**. The figure shows changes in circumferential and radial strain in respect to changes of left ventricular ejection fraction (EF) at rest and with dobutamine stress (10 and 20 μg/kg^-1^/min^-1^). Values expressed as mean with standard deviation. *LV = left ventricle, EF = ejection fraction*.

Changes in Ecc_SAX _and Err_SAX _were significant between rest and both levels of dobutamine as well as between 10 and 20 μg · kg^-1^· min^-1 ^of dobutamine (table 2; table 3; figure 2). In parallel with these changes LV-EF increased significantly with 10 and 20 μg · kg^-1^· min^-1 ^of dobutamine (table 2; figure 2).

**Table 2:** The hemodynamic response and the response in strain parameters to 10 and 20 μg · kg^-1^· min^-1 ^of dobutamine

Parameter	Level of Dobutamine (μg/kg^-1^/min^-1^)			Significance: Paired t-test		
	**Rest**	**10**	**20**	**Rest-10**	**Rest-20**	**10-20**

**Heart Rate (bpm)**	68.6 ± 11.9	87.1 ± 15.5	115.7 ± 11.1	<0.05	<0.05	<0.05
**Mean BP (mmHg)**	91.5 ± 10.2	98.6 ± 10.4	102.9 ± 10.7	<0.05	<0.05	<0.05
**LV-CI (l/min/m^2^)**	3.0 ± 0.6	4.8 ± 0.8	5.7 ± 0.8	<0.05	<0.05	<0.05
**RV-CI (l/min/m^2^)**	3.0 ± 0.6	4.7 ± 0.8	5.8 ± 4.0	<0.05	<0.05	<0.05
**LV-EDV (ml/m^2^)**	76.9 ± 12.5	75.0 ± 12.5	64.5 ± 11.5	0.25	<0.05	<0.05
**LV-ESV (ml/m^2^)**	33.4 ± 7.5	22.1 ± 7.3	15.2 ± 5.0	<0.05	<0.05	<0.05
**LV-SV (ml/m^2^)**	43.5 ± 6.5	52.9 ± 10.6	49.3 ± 8.0	<0.05	<0.05	0.06
**LV-EF (%)**	56.9 ± 4.4	70.7 ± 8.1	76.8 ± 4.6	<0.05	<0.05	<0.05
**Err_SAX_**	19.6 ± 14.6	31.8 ± 20.9	42.4 ± 25.5	<0.05	<0.05	<0.05
**Ecc_SAX_**	-24.1 ± 6.7	-32.7 ± 11.4	-39.2 ± 15.2	<0.05	<0.05	<0.05

The table shows the hemodynamic response and the response in strain parameters to 10 and 20 μg · kg^-1^· min^-1 ^of dobutamine. Volumetric values were indexed for body surface area and expressed as mean ± standard deviation. Paired t-test was used to determine the significance of change from one level of dobutamine to next (p < 0.05). *LV = left ventricle SV = stroke volume ESV = end-systolic volume EDV = end-diastolic volume, EF = ventricular ejection fraction, Err_SAX_ = left ventricular short-axis radial strain; Ecc_SAX_ = left ventricular short-axis circumferential strain*

**Table 3:** The response in strain parameters to 10 and 20 μg · kg^-1^· min^-1 ^of dobutamine on a segmental basis

Parameter	Level of Dobutamine (μg/kg^-1^/min^-1^)			Significance: Paired t-test		
	**Rest**	**10**	**20**	**Rest-10**	**Rest-20**	**10-20**

**Err_SAX _Average**	19.6 ± 14.6	31.8 ± 20.9	42.4 ± 25.5	<0.05	<0.05	<0.05
**Err_SAX _Segment 7**	27.3 ± 16	33.1 ± 20	50.2 ± 29	0.4	<0.05	0.1
**Err_SAX _Segment 8**	13 ± 9.5	22.6 ± 11.5	28.2 ± 15.2	<0.05	<0.05	0.2
**Err_SAX _Segment 9**	11.1 ± 9.9	17.6 ± 13.4	22 ± 17	0.08	<0.05	0.3
**Err_SAX _Segment 10**	16.8 ± 12	31.7 ± 20.3	37.2 ± 19.2	<0.05	<0.05	0.22
**Err_SAX _Segment 11**	21.6 ± 15.9	42.7 ± 20	55.8 ± 19.1	<0.05	<0.05	<0.05
**Err_SAX _Segment 12**	27.7 ± 16.1	43.4 ± 26.6	60.7 ± 28.1	0.1	<0.05	0.18
**Ecc_SAX _Average**	-24.1 ± 6.7	-32.7 ± 11.4	-39.2 ± 15.2	<0.05	<0.05	<0.05
**Ecc_SAX _Segment 7**	-21.7 ± 6.9	-24.2 ± 6.3	-28.9 ± 16	0.29	0.2	0.2
**Ecc_SAX _Segment 8**	-20.2 ± 8.2	-25.9 ± 12.4	-33.9 ± 18	0.06	<0.05	0.09
**Ecc_SAX _Segment 9**	-22.8 ± 6.9	-35.7 ± 9	-42.1 ± 9.8	<0.05	<0.05	0.1
**Ecc_SAX _Segment 10**	-26.7 ± 5.5	-36.6 ± 8.8	-38.8 ± 16.3	0.09	0.06	0.55
**Ecc_SAX _Segment 11**	-29.1 ± 4.7	-38.1 ± 13.2	-47 ± 9.5	<0.05	<0.05	0.09
**Ecc_SAX _Segment 12**	-23.8 ± 4.3	-35.9 ± 10.9	-44.2 ± 14.9	0.09	<0.05	0.06

The table shows the response in strain parameters to 10 and 20 μg · kg^-1^· min^-1 ^of dobutamine on a segmental basis derived from mid-ventricular short-axis view containing both papillary muscles. Values are expressed as mean ± standard deviation. Paired t-test was used to determine the significance of change from one level of dobutamine to the next one (p < 0.05). *Err_SAX_ = left ventricular short-axis radial strain; Ecc_SAX_ = left ventricular short-axis circumferential strain*

### Intra- and inter-observer variability

**Figure 3:**
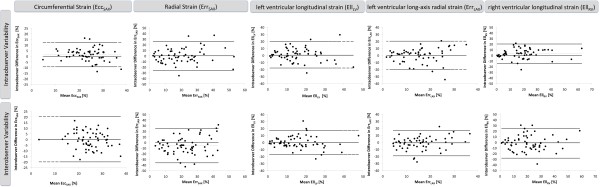
**Bland Altman Plots for intra- and inter-observer variability**. Bland Altman Plots for intra- and inter-observer variability obtained for all strain parameters at rest on a segmental basis

All parameters were reproducible on an intra- and inter-observer level. Bland Altman plots are displayed in figure 3 and table 4 shows the 95% confidence intervals of the difference between the repeated measurements. Observer variability at rest was best for Ecc_SAX _and worst for Ell_RV _as determined by 95% confidence intervals of the difference. Observer variability did not significantly increase with dobutamine stress (table 5).

**Table 4:** Intra- and inter-observer variability of different strain parameters

Parameter	Ventricle	Variability	Mean	CI (95%)	p-value
**Ecc_SAX_**	LV	Intra-observer	24.1	22.3-25.8	0.06
			22.7	20.8-24.6	
		Inter-observer	24.1	22.3-25.8	0.61
			24.6	22.6-26.6	

**Err_SAX_**	LV	Intra-observer	19.6	15.8-23.4	0.86
			19.9	16.5-23.2	
		Inter-observer	19.6	15.8-23.4	0.06
			25.4	22.3-28.4	

**Err_LAX_**	LV	Intra-observer	15.3	12.7-18	1
			15.3	13-17.7	
		Inter-observer	15.3	12.7-18	0.32
			16.6	14.4-18.7	

**Ell_LV_**	LV	Intra-observer	15.9	13.2-18.6	0.57
			15.2	12.4-18.1	
		Inter-observer	15.9	13.2-18.6	0.82
			16.2	13.2-19.1	

**Ell_RV_**	RV	Intra-observer	19.6	16-23.3	0.13
			16.8	13.4-20.1	
		Inter-observer	19.6	16-23.3	0.32
			21.4	17.8-25	

The table shows intra- and inter-observer variability of different strain parameters. 95% Confidence Intervalls of the difference and p-values are given to accurately determine individual variabilities [12]. *Ecc_SAX_ = left ventricular short-axis circumferential strain, Err_SAX_ = left ventricular short-axis radial strain, Err_LAX_ = left ventricular long-axis radial strain, Ell_LV_ = left ventricular longitudinal strain, Ell_RV_ = right ventricular longitudinal strain, LV = left ventricle, RV = right ventricle.*

**Table 5:** Intra- and inter-observer variability of circumferential and radial strain parameters of the LV at rest and with dobutamine stress

Parameter	Ventricle	Variability	Mean	CI (95%)	p-value
**Ecc_SAX_**	LV	Intra-observer	24.1	22.3-25.8	0.06
			22.7	20.8-24.6	
		Inter-observer	24.1	22.3-25.8	0.61
			24.6	22.6-26.6	

**Ecc_SAX10_**	LV	Intra-observer	32.7	29.8-35.8	0.09
			31.1	28.3-34.9	
		Inter-observer	32.7	29.8-35.8	0.66
			33.4	29.9-37	

**Ecc_SAX20_**	LV	Intra-observer	39.2	35.2-43.1	0.25
			41	37.9-43.9	
		Inter-observer	39.2	35.2-43.1	0.17
			41.2	37.8-44.6	

**Err_SAX_**	LV	Intra-observer	19.6	15.8-23.4	0.86
			19.9	16.5-23.2	
		Inter-observer	19.6	15.8-23.4	0.06
			25.4	22.3-28.4	

**Err_SAX10_**	LV	Intra-observer	31.8	26.9-37.9	0.2
			34.9	29.9-39.8	
		Inter-observer	31.8	26.9-37.9	0.14
			35.5	30.7-40.3	

**Err_SAX20_**	LV	Intra-observer	42.4	35.8-48.9	0.31
			44.9	36.2-49.1	
		Inter-observer	42.4	35.8-48.9	0.59
			43.5	38.4-48.5	

The table shows intra- and inter-observer variability of circumferential and radial strain parameters of the LV derived from a short-axis view at rest and with dobutamine stress (10 and 20 μg · kg^-1^· min^-1^). 95% Confidence Intervalls of the difference and p-values are given to accurately determine individual variabilities [12]. *Ecc_SAX_ = left ventricular short-axis circumferential strain, Err_SAX_ = left ventricular short-axis radial strain,*

### Comparison with natural radial strain

**Figure 4:**
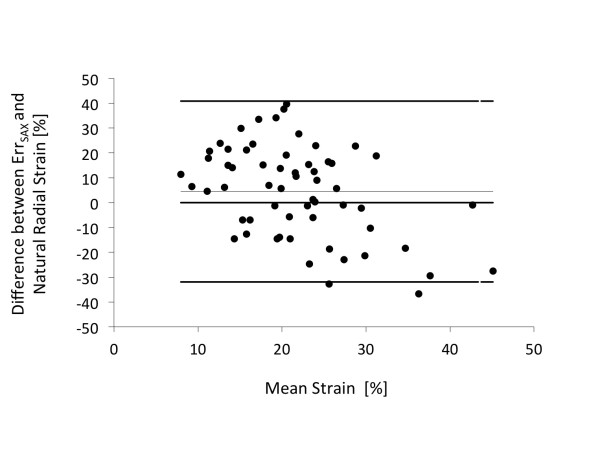
**Bland Altman Plot showing the relationship between Err_SAX _and natural radial strain**. Bland Altman Plot showing the relationship between Err_SAX _(Mean 19.6; 15.8-23.4 95% confidence interval) and natural radial strain (Mean 24; 21.7-26.4 95% confidence interval). *Err_SAX _= left ventricular short-axis radial strain*

There was reasonable agreement between mean Err_SAX _and natural radial strain as demonstrated in figure 4.

## Discussion

The current study includes a unique population of healthy volunteers studied at rest and with DS-CMR and demonstrates several important findings.

Firstly; CMR-FT can quantify wall motion changes between rest and dobutamine stress. Secondly; we noted considerable intra- and inter-observer variability for all parameters; which was most pronounced for RV longitudinal strain and smallest for LV circumferential strain. Thirdly; normal values of CMR-FT cover a large range with considerable overlap between rest and stress between different subjects (Figure 2) and closely correlate with hemodynamic changes secondary to changed LV function.

In essence; our data demonstrate that CMR-FT strain parameters can be derived from routine SFFP cine sequences at varying levels of DS-CMR. The current reference standard for quantitative wall motion assessment with CMR is myocardial tagging [11,14,15]. Myocardial tagging based strain assessment has been demonstrated to improve diagnostic accuracy of DS-CMR in patients with suspected CAD as well as in patients with myocardial hibernation [16]. Adding quantitative analysis to DS-CMR in CAD may not only increase diagnostic accuracy as compared to visual analysis for the detection of ischemia during high-dose dobutamine stress [17] but may also detect quantitative changes in myocardial strain already detectable at lower stress levels; thereby increasing feasibility of the test [18].

There is evidence that CMR-FT may also be of clinical utility. Hor et al have recently shown that CMR-FT provides similar results compared to HARP myocardial tagging in a patient population with Duchenne muscular dystrophy [5]. Maret et al demonstrated that CMR-FT can be used in CAD patients to accurately detect strain in the radial and longitudinal direction correlating with the presence of myocardial scarring [4]. CMR-FT is also useful for the assessment of myocardial viability [19]. Detection of quantitative contractile reserve in patients with myocardial hibernation using low dose dobutamine has the potential to predict functional recovery after revascularisation with higher accuracy in the future [20]. In this context; CMR-FT may serve as an additional tool alongside conventional visual analysis; thus facilitating the detection of subtle wall motion abnormalities and the identification of contractile reserve; particularly for the less experienced observer.

The importance of the intra- and interobserver variability documented in the current study needs to be taken into consideration. This has also been reported by echocardiography based speckle tracking studies and our results are similar [21]. The parameter with highest variability at rest in the current study was longitudinal strain of the RV indicating that the analysis of the thin-walled RV with CMR-FT is not yet adequately accurate. This might also be explained by difficulties in endocardial tracking due to difficulty in accurately following the tricuspid valve annulus motion with the CMR-FT software; and RV trabeculations that also lead to greater variability in RV volumetric assessment [22]. The most robust parameter in our study was circumferential strain of the LV; which might be clinically valuable. CMR- FT algorithm allows reliable and easy border tracking; the frame-to-frame displacement of features tracked is equivalent to evaluating the local velocity (ratio between displacement and time interval); allowing automatic evaluation of tissue motion during the cardiac cycle. The tracking results from this algorithm may be more reliable due to the inherently high image quality with CMR. However echocardiographic speckle tracking has better temporal resolution than CMR-FT. In addition our collective consisted of good breath holders and as a consequence we obtained good image quality at an intermediate stress level. It is therefore not surprising that observer-induced variance did not significantly increase with stress. Whether potentially degraded image quality at higher stress levels in patients who are not able to hold their breath would substantially obscure CMR-FT results needs to be prospectively assessed. Interestingly results in radial strain from matching segments from the short-axis and long-axis orientation were not equal. Whether this could be explained by more extensive through-plane motion in the short-axis orientation or increased susceptibility of the 4-chamber view to the breath-holding position of the diaphragm needs to be investigated in healthy volunteers and in patients with scarred areas and segments with wall motion abnormalities. In particular future studies need to investigate whether DS-CMR accuracy could be improved with CMR-FT information that is available with any DS-CMR stress study. There is evidence to suggest that these quantitative parameters have prognostic implications. Stanton and colleagues demonstrated that automated echocardiography speckle-tracking derived global Ell_LV _is a superior predictor of outcome compared to either EF or wall motion score index; and suggested that Ell_LV _may even become the optimal method to assess global left ventricular systolic function [23]. As CMR-FT is a relatively new method such evidence is not yet available and future studies need to investigate whether CMR-FT could also provide such assessment.

### Limitations

The sample size of the current study was relatively small. Future studies will need to reassess these parameters in a larger cohort of volunteers and patients. Global Ell; which has been previously shown to be an important; prognostic echocardiographic parameter was only assessed at rest. This was due to time constraints as a whole stack of short axis images had been acquired at each stage of dobutamine for volumetry. Also we did not perform any echocardiographic measurements to compare with CMR-FT data; which needs to be addressed in future studies. Finally the current work aimed to determine the feasibility of CMR-FT during dobutamine stress in a collective of healthy volunteers. Future research needs to prospectively validate this novel technique in pathologies such as coronary artery disease; valvular disease or congenital disorders.

## Conclusions

CMR-FT allows derivation of strain mechanics from SSFP cine images at rest and during dobutamine stress CMR corresponding to global hemodynamic changes. The current analysis algorithm requires improvement to reduce observer-induced variance; which at present is comparable to data reported from 2D strain by echocardiography. Within a given CMR lab; this novel CMR-FT technique holds promise for easy and fast quantification of wall mechanics and strain.

## Abbreviations

AMI: acute myocardial infarction; CMR: cardiovascular magnetic resonance; DS-CMR: dobutamine stress cardiovascular magnetic resonance; Ecc_SAX_: left ventricular short-axis circumferential strain; EDV: enddiastolic volume; EF: ejection fraction; Ell_LV_: left ventricular longitudinal strain; Ell_RV_: right ventricular longitudinal strain; EDWT: end-diastolic wall thickness; ESWT: end-systolic wall-thickness; Err_LAX_: left ventricular long-axis radial strain; Err_SAX_: left ventricular short-axis radial strain; ESV: endsystolic volume; FT: myocardial feature tracking; LV: left ventricle; RV: right ventricle; SV: stroke volume.

## Competing interests

The authors declare that they have no competing interests.

## Authors' contributions

AS and SK designed the study protocol; analyzed the data and drafted the manuscript. AP; PG and DAD performed the CMR studies and helped to draft the manuscript. VP and MRM participated in the study design and helped to draft the manuscript. BB participated in the study design and helped with the statistical analysis. PB and EN designed the study protocol and drafted the manuscript. All authors read and approved the final manuscript.

## References

[B1] Morton G Schuster A Perera D Nagel E Cardiac magnetic resonance imaging to guide complex revascularization in stable coronary artery disease European heart journal 2010 31 2209 2215 10.1093/eurheartj/ehq256 20705696

[B2] Paetsch I Jahnke C Ferrari VA Rademakers FE Pellikka PA Hundley WG Poldermans D Bax JJ Wegscheider K Fleck E Nagel E Determination of interobserver variability for identifying inducible left ventricular wall motion abnormalities during dobutamine stress magnetic resonance imaging Eur Heart J 2006 27 1459 1464 16613929 10.1093/eurheartj/ehi883

[B3] Zerhouni EA Parish DM Rogers WJ Yang A Shapiro EP Human heart: tagging with MR imaging--a method for noninvasive assessment of myocardial motion Radiology 1988 169 59 63 3420283 10.1148/radiology.169.1.3420283

[B4] Maret E Todt T Brudin L Nylander E Swahn E Ohlsson JL Engvall JE Functional measurements based on feature tracking of cine magnetic resonance images identify left ventricular segments with myocardial scar Cardiovasc Ultrasound 2009 7 53 10.1186/1476-7120-7-53 19917130 PMC2785780

[B5] Hor KN Gottliebson WM Carson C Wash E Cnota J Fleck R Wansapura J Klimeczek P Al-Khalidi HR Chung ES Comparison of magnetic resonance feature tracking for strain calculation with harmonic phase imaging analysis JACC Cardiovasc Imaging 2010 3 144 151 10.1016/j.jcmg.2009.11.006 20159640

[B6] Schuster A Nagel E Toward Full Quantification of Wall Motion with MRIcurr cardiovasc imaging rep 2011 85 86 10.1007/s12410-011-9072-x

[B7] Nagel E Lehmkuhl HB Bocksch W Klein C Vogel U Frantz E Ellmer A Dreysse S Fleck E Noninvasive diagnosis of ischemia-induced wall motion abnormalities with the use of high-dose dobutamine stress MRI: comparison with dobutamine stress echocardiography Circulation 1999 99 763 770 9989961 10.1161/01.cir.99.6.763

[B8] Thiele H Paetsch I Schnackenburg B Bornstedt A Grebe O Wellnhofer E Schuler G Fleck E Nagel E Improved accuracy of quantitative assessment of left ventricular volume and ejection fraction by geometric models with steady-state free precession Journal of cardiovascular magnetic resonance: official journal of the Society for Cardiovascular Magnetic Resonance 2002 4 327 339 10.1081/JCMR-120013298 12234104

[B9] Cerqueira MD Weissman NJ Dilsizian V Jacobs AK Kaul S Laskey WK Pennell DJ Rumberger JA Ryan T Verani MS Imaging AHAWGoMSaRfC Standardized myocardial segmentation and nomenclature for tomographic imaging of the heart: a statement for healthcare professionals from the Cardiac Imaging Committee of the Council on Clinical Cardiology of the American Heart Association Circulation 2002 105 539 542 10.1161/hc0402.102975 11815441

[B10] Mirsky I Parmley WW Assessment of passive elastic stiffness for isolated heart muscle and the intact heart Circulation Research 1973 33 233 243 4269516 10.1161/01.res.33.2.233

[B11] Attili AK Schuster A Nagel E Reiber JHC van der Geest RJ Quantification in cardiac MRI: advances in image acquisition and processing The international journal of cardiovascular imaging 2010 26 Suppl 1 27 40 20058082 10.1007/s10554-009-9571-x PMC2816803

[B12] Bland JM Altman DG Statistical methods for assessing agreement between two methods of clinical measurement Lancet 1986 1 307 310 2868172

[B13] Hudsmith LE Petersen SE Francis JM Robson MD Neubauer S Normal human left and right ventricular and left atrial dimensions using steady state free precession magnetic resonance imaging Journal of cardiovascular magnetic resonance: official journal of the Society for Cardiovascular Magnetic Resonance 2005 7 775 782 10.1080/10976640500295516 16353438

[B14] Shehata ML Cheng S Osman NF Bluemke DA Lima JAC Myocardial tissue tagging with cardiovascular magnetic resonance Journal of cardiovascular magnetic resonance: official journal of the Society for Cardiovascular Magnetic Resonance 2009 11 55 10.1186/1532-429X-11-55 PMC2809051 20025732

[B15] Ibrahim E-SH Myocardial tagging by Cardiovascular Magnetic Resonance: evolution of techniques--pulse sequences, analysis algorithms, and applications Journal of cardiovascular magnetic resonance: official journal of the Society for Cardiovascular Magnetic Resonance 2011 13 36 10.1186/1532-429X-13-36 PMC3166900 21798021

[B16] Nagel E Schuster A Shortening without contraction: new insights into hibernating myocardium JACC Cardiovasc Imaging 2010 3 731 733 10.1016/j.jcmg.2010.05.002 20633851

[B17] Kuijpers D Ho KY van Dijkman PR Vliegenthart R Oudkerk M Dobutamine cardiovascular magnetic resonance for the detection of myocardial ischemia with the use of myocardial tagging Circulation 2003 107 1592 1597 10.1161/01.CIR.0000060544.41744.7C 12668491

[B18] Korosoglou G Lehrke S Wochele A Hoerig B Lossnitzer D Steen H Giannitsis E Osman NF Katus HA Strain-encoded CMR for the detection of inducible ischemia during intermediate stress JACC Cardiovasc Imaging 2010 3 361 371 10.1016/j.jcmg.2009.11.015 20394897

[B19] Schuster A Paul M Bettencourt N Morton G Chiribiri A Ishida M Hussain S Jogiya R Kutty S Bigalke B Cardiovascular magnetic resonance myocardial feature tracking for quantitative viability assessment in ischemic cardiomyopathy. International Journal of Cardiology 2011 [E-pub ahead of print], doi:10.1016/j.ijcard.2011.10.137 10.1016/j.ijcard.2011.10.137 22130224

[B20] Bree D Wollmuth JR Cupps BP Krock MD Howells A Rogers J Moazami N Pasque MK Low-dose dobutamine tissue-tagged magnetic resonance imaging with 3-dimensional strain analysis allows assessment of myocardial viability in patients with ischemic cardiomyopathy Circulation 2006 114 I33 36 10.1161/CIRCULATIONAHA.105.600858 16820595 PMC1501089

[B21] Bansal M Jeffriess L Leano R Mundy J Marwick TH Assessment of myocardial viability at dobutamine echocardiography by deformation analysis using tissue velocity and speckle-tracking JACC Cardiovasc Imaging 2010 3 121 131 10.1016/j.jcmg.2009.09.025 20159637

[B22] Beerbaum P Barth P Kropf S Sarikouch S Kelter-Kloepping A Franke D Gutberlet M Kuehne T Cardiac function by MRI in congenital heart disease: impact of consensus training on interinstitutional variance J Magn Reson Imaging 2009 30 956 966 10.1002/jmri.21948 19856409

[B23] Stanton T Leano R Marwick TH Prediction of all-cause mortality from global longitudinal speckle strain: comparison with ejection fraction and wall motion scoring Circ Cardiovasc Imaging 2009 2 356 364 19808623 10.1161/CIRCIMAGING.109.862334

